# NUPR1 packaged in extracellular vesicles promotes murine triple-negative breast cancer in a type 1 interferon-independent manner

**DOI:** 10.20517/evcna.2023.59

**Published:** 2024-01-15

**Authors:** Angelica Ortiz, Aikaterini Stavrou, Shan Liu, Danqi Chen, Steven S. Shen, Chunyuan Jin

**Affiliations:** 1 Department of Medicine, Division of Environmental Medicine, New York University Grossman School of Medicine, New York, NY 10010, USA.; 2 Department of Biomedical Science, School of Veterinary Medicine, University of Pennsylvania, Philadelphia, PA 19104, USA.; 3 Clinical Translational Science Institute, University of Minnesota, Minneapolis, MN 55455, USA.

**Keywords:** Extracellular vesicles, NUPR1, triple-negative breast cancer, chemotherapy, type 1 interferon signaling

## Abstract

**Aim:**

This study aims to elucidate the involvement of triple-negative breast cancer (TNBC)-derived extracellular vesicles in metastasis. The loss of components in the type 1 interferon (IFN1) signaling pathway has been linked to the promotion of metastasis. However, IFN1 signaling induces immunological dormancy and promotes tumorigenesis. Our hypothesis was that TNBC cells release tumor-derived extracellular vesicles (TEVs) that promote metastasis in an IFN1-independent manner.

**Methods:**

Two murine TNBC models and transgenic mice were used to examine the role of IFN1 in TNBC progression to metastasis. Reserpine was employed to determine the effect of TEV education on TNBC progression and overall survival. EVs from cancer cells treated with vehicle and reserpine and from the serum of tumor-bearing mice receiving reserpine were examined to determine changes in EV release and EV content.

**Results:**

TNBC cells progress to metastasis in mice lacking the IFN1-induced gene cholesterol-25 hydroxylase (CH25H) or expressing the IFNAR1^S526^ knock-in that cannot be downregulated. Reserpine suppresses EV release from TNBC cells *in vitro* and *in vivo*. Western blot analysis demonstrated reserpine decreased NUPR1 protein levels in EVs. RNAseq analysis demonstrated that endothelial cells lacking CH25H treated with TEVs exhibited increased NUPR1 expression that was decreased by adding reserpine with the TEVs. NUPR1 overexpression upregulated genes that mediate TEV biogenesis and incorporation. Knockdown of NUPR1 with shRNA decreased the release of TEVs.

**Conclusion:**

In conclusion, our study suggests that TNBC is driven by aberrant packaging of NUPR1 into TEVs which were transferred into recipient cells to activate pro-metastatic transcription driven by NUPR1.

## INTRODUCTION

Triple-negative breast cancer (TNBC) is classified as an aggressive form of breast cancer. TNBC patients are at higher risk to develop recurrence and/or metastasis^[1,2]^. The development of distal metastasis greatly contributes to cancer-related death. Therefore, identifying mechanisms that govern TNBC progression is of great importance. The lack of receptors for human epidermal growth factor, progesterone, and estrogen in TNBC cells makes hormonal therapy ineffective^[1–3]^. Cytotoxic drugs, such as paclitaxel (PTX), have proven effective early in TNBC treatment^[1–4]^. However, TNBC patients develop resistance following multiple cycles with chemotherapy to further limit therapeutic intervention and increase the risk of mortality. Preclinical studies have demonstrated that PTX treatment in TNBC tumor-bearing mice increased TNBC cells in circulation, resulting in increased lung metastasis^[5,6]^. A study demonstrated that TNBC tumor-bearing mice treated with PTX developed more pulmonary metastatic lesions than mice treated with vehicle^[5]^.

Type 1 interferon (IFN1) signaling induces the expression of genes that participate in anti-viral, anti-proliferative, and anti-tumorigenic functions^[7–14]^. Studies have demonstrated in murine mouse models that loss of type 1 interferon (IFN1) signaling accelerated breast cancer progression and increased incidence of metastasis^[7,14]^. However, there is also evidence that IFN1 signaling induces immunological dormancy following chemotherapy^[15]^ and promotes tumorigenesis of TNBC^[16]^. Moreover, unphosphorylated signal transducer and activator of transcription 1 (STAT1) may sustain the expression of interferon-stimulated genes (ISGs) that contribute to resistance to DNA damage, which may contribute to diminished response to cancer therapy^[9]^. Therefore, it is important to identify mechanisms that contribute to TNBC progression to overcome complete loss or hyperactive IFN1 signaling. In a previous study, we demonstrated that cancer cell-derived EVs (TEVs) from melanoma cells and melanoma patients downregulated the type 1 interferon receptor subunit 1 (IFNAR1), resulting in loss of the interferon-stimulated gene (ISG) cholesterol-25 hydroxylase (CH25H)^[13]^. We demonstrated that reserpine, a discontinued blood pressure medication, suppressed EV incorporation and prevented cancer cell EV-mediated cancer progression by suppressing the loss of IFNAR1 and CH25H^[13]^. Moreover, we demonstrated that administration of conventional cancer therapies (ionizing radiation or chemotherapy) in combination with reserpine delayed tumor growth and suppressed cancer progression to improve overall survival^[12]^. QPCR analysis demonstrated altered expression of tetraspanins that mediate EV incorporation^[12]^. However, how reserpine affects the transcription of these tetraspanins remains unclear. Therefore, we must develop interventions that target EV incorporation and release of EVs that advance cancer progression. To achieve this, we must identify the transcription factors that regulate the expression of proteins involved in governing EV biogenesis and incorporation.

Nuclear protein 1 (NUPR1), also called p8, is a stress-activated transcription factor that regulates cellular stress response^[17–21]^. In cancer, NUPR1 expression and transcriptional activity are increased^[17–21]^. The elevated expression and function of NUPR1 contribute to increased cancer cell migration, invasion, development of metastatic lesions, and resistance to cancer therapies^[17–21]^. Various breast cancer cell lines that were classified as positive for estrogen receptor (ER+) and TNBC demonstrated elevated NUPR1 mRNA and protein compared to non-breast cancer cells^[20]^. Immunohistochemistry analysis also demonstrated increased NUPR1 protein levels in breast tumor tissue in comparison to normal adjacent tissue^[20]^. Interestingly, tamoxifen, a common chemotherapy agent, induced the expression of NUPR1, and the subsequent expression of NUPR1 conferred resistance to tamoxifen therapy^[20]^. Tamoxifen treatment induced breast cancer cells to release EVs that conferred drug resistance^[20]^. We posited that TNBC progression was independent of the IFN1 signaling but was mediated through NUPR1 packaged within cancer cell-derived EVs and that reserpine functioned in limiting NUPR1 packaging into EVs and suppressed NUPR1 expression in recipient cells treated with EVs.

Here, we present data demonstrating that while loss of the ISG CH25H and overactive IFN1 signaling promote TNBC progression to lung metastasis, reserpine is able to suppress TEV-induced cancer progression by downregulation of NUPR1 in TEV recipient cells and decrease NUPR1 in TEVs released by reserpine treated cancer cells.

## MATERIALS AND METHODS

### Animal studies

All experiments with animals were carried out under protocol 803995 approved by the Institutional Animal Care and Use Committee (IACUC) of The University of Pennsylvania. *Ch25h*^−/−^ mice were obtained from the Jackson Laboratory. *Ch25h*^−/−^ mice were of C57BL/6 and wild-type mice were either of C57BL/6 or Balb/c background. The Ifnar1^S526A^ (SA) mice were backcrossed ten times into the Balb/c background. All mice were provided water and chow ad libitum. Mice were housed in a pathogen-free facility in accordance with American Association for Laboratory Animal Science guidelines. Experimental groups were set up using littermates that were randomly assigned and then were either co-housed or systematically exposed to other groups’ bedding. This setup was necessary to ensure equal exposure to all group’s microbiota. Only female mice of 5 to 7 weeks of age were used, as breast cancer is predominantly associated with females.

### Cell culture

Mouse mammary adenocarcinoma EO7771 cell line was purchased from CH3 Biosystems and cultured according to this provider’s recommendations. The 4T1-GFP cell line was provided by Dr. Rumela Chakrabarti (University of Pennsylvania) and was cultured in RPMI-1640 media with 10% extracellular vesicle-free (EV-free) fetal bovine serum (FBS). Human bronchial epithelial cells BEAS-2B were obtained from ATCC (CRL-9609, ATCC, Manassas, VA, USA). BEAS-2B cells were cultured to the specifications of the vendor’s recommendation, which includes Bronchial Epithelial Cell Growth Medium (BEGM, Lonza, Walkersville, MD, USA) that was supplemented with 1% penicillin-streptomycin (GIBCO, Grand Island, NY, USA). The nickel-transformed BEAS-2B cells expressing shRNA with scramble sequence or NUPR1 were also provided by Dr. Max Costa^[22]^. Nickel-transformed cells were grown in DMEM with 10% EV-free FBS. MC38 murine colon adenocarcinoma was purchased from Millipore Sigma and cultured in DMEM with 10% EV-free FBS.

### Cell transfection

For NUPR1 overexpression, the pcDNA3.1-FLAG-Nupr1 plasmid was transfected into BEAS-2B cells using PolyJet (SignaGen), according to the manufacturer’s instructions. The NUPR1 cDNA with a flag tag was purchased from Origene (RC22237). The pcDNA3.1-FLAG-Empty vector was used as the control.

### RNA sequencing

Complementary DNA (cDNA) libraries were generated from the RNA of BEAS-2B cells expressing empty vector or NUPR1 overexpression vector using a TruSeq RNA Sample Preparation V2 Kit (Illumina). Library preparations were validated with the Agilent Bioanalyzer using the DNA1000 kit. We adjusted the library concentrations to 4 nM. The libraries were then pooled for multiplex sequencing. Pooled libraries were denatured and then diluted to 15 pM. The diluted libraries were clonally clustered onto the sequencing flow cell using the Illumina cBOT Cluster Generation Station and a TruSeq Paired-End Cluster Kit v3-cBot-HS. We performed the sequencing using an Illumina HiSeq2500 Sequencing System using a TruSeq SBS Kit v3-HS. Raw sequencing reads were mapped to the human genome reference (GRCh37.71/hg19) using Bowtie aligner (0.12.9) with v2 and m1 parameters. Mapped reads were subsequently subjected to PCR duplicate removal prior to gene model assignment using featureCounts package. To identify significant differentially expressed genes, the raw reads of each sample from experimental and control groups were normalized and compared based on experimental design using default methods with the edgeR package (3.4.2). The FDR adjustment was employed for multiple hypothesis tests, and an appropriate FDR cutoff was applied to select significant differentially expressed genes for analysis.

### Extracellular vesicle isolation from cell culture and serum

The TEVs were separated using the ultracentrifugation method as previously described^[13,23]^. Briefly, cells were cultured in the appropriate culture media that was free of FBS EVs. The conditioned media was collected from plates that were no more than 80% confluent. To remove live cells, medium was centrifuged at 2000× *g* for 30 min. The supernatant was then centrifuged at 10,000 rpm for 15 min using the SW32Ti rotor in the Beckman Coulter Optima XL-100K ultracentrifuge at 4 °C to remove dead cells and cell debris. To collect the EVs, the supernatant medium was then centrifuged at 25,000 rpm overnight at 4 °C. The EV pellet was resuspended and washed in PBS for the last centrifugation at 25,000 rpm at 4 °C for 3 h. The PBS wash is discarded and washed EV pellet is resuspended in PBS. EVs were stored at −80 °C for long-term storage and thawed on ice before use. EVs were characterized by diameter and particle number by the Nanomedicines Characterization Core Facility in the University of North Carolina at Chapel Hill using the NanoSight NS500 (Malvern Panalytical, Ltd, UK) and confirmed using the Zetaview QUATT (Particle Metrix, Ammersee, Germany) in the Flow Cytometry core facility at NYU Langone Health Center. For transmission electron microscopy (TEM), EVs were diluted 1:50 in 0.1 μm filtered PBS PBS (Corning, NY, USA). A volume of 4 μL of EV sample was placed onto the surface of a 400 mesh carbon film-coated grid (SKU CF400-Cu-50 by EMS, Hatfield, PA). The sample was immediately removed from the grid using filter paper. Without letting the grid dry, 4 μL of 0.75% uranyl formate was added to the grid and removed immediately through filtration. This wash with 0.75% uranyl formate was repeated three times. The third time, 0.75% uranyl formate was left on the grid for 5 min to stain the samples. After 5 min, 0.75% uranyl formate was removed using a filter paper and the grid was left to dry for 5 min at room temperature (RT) before visualization was performed. Samples were imaged at RT using JEOL1400 Flash TEM microscope with GATAN 4k × 4k Rio CMOS camera (JOEL LTD, Japan). The grids were analyzed under 100 kV and images were analyzed via TEMography^™^ (JOEL LTD, Japan). Images were taken at a 25,000x magnification. We determined the protein concentration of EVs using the Pierce BCA Protein Assay Kit. Extracellular vesicles were stored at −80 °C for long-term storage and thawed on ice before use.

To isolate EVs from serum, 110 μL of serum from mice with tumors of similar size was centrifuged at 2,000× *g* for 30 min and 100 μL of the cleared serum was incubated with 20 μL Total Exosome Isolation Reagent from Serum (Invitrogen, Waltham, MA), which ties up water molecules allowing the EVs to be collected by low-speed centrifugation.

### Isolation of primary mouse splenocytes and peripheral blood leukocytes

Spleens were collected from 3–5 week-old mice immediately following euthanasia. The spleens were forcibly traversed through a 70 μm cell strainer and washed with ice-cold PBS. Red blood cell lysis buffer was added to the samples and then washed three times with PBS before being prepped for EV treatments or flow cytometry experiments.

Peripheral blood samples were collected aseptically from the mouse tail vein or cardiac puncture into (green top) Na Heparin Capiject tubes. The blood samples were suspended in red blood cell lysis buffer and washed thoroughly with PBS prior to experimental use.

### Lung tissue dissociation

Shortly after euthanasia, we collected lung tissues and washed them with ice-cold PBS. We then cut the lung tissue into smaller pieces using sterile, sharp dissection scissors. This tissue homogenate was then incubated in dissociation solution (3 mL/lung) (dissociation solution: 2 mg/mL Collagenase II, 1 mg/mL Collagenase D plus 100 μg/mL Dnase I solution) for approximately 1 hr in a sterile glass vial and a magnetic stir bar providing continuous agitation at 37 °C. We then passed this digested tissue through 70 μm cell strainers before quenching the digestion reaction using 10 mL of 10% FBS RPMI medium. We then incubated the cells with RBC lysis buffer for 5 min to remove red blood cells and washed the cells three times with PBS before flow cytometry experiments.

### Flow cytometry

Following washing with PBS, the mouse cells were incubated with mouse Fc block in blocking solution (blocking solution: 0.5% BSA-PBS). Cells were then incubated with anti-mouse CD45 conjugated with APC and anti-mouse IFNAR1 conjugated with PE. We washed the cells with PBS to remove unbound antibodies and then resuspended the cells in PBS with DAPI for flow cytometry analysis on FACS Canto II and LSR Fortessa (BD Biosciences) and the FACS DIVA software. In all cases, the changes in mean fluorescence intensity (ΔMFI) were calculated as follows:

ΔMFI=SampleMFI−Control(IgG)MFI

### In vivo mammary tumor growth

EO771 and 4T1-GFP cells were lifted using 2mM EDTA in PBS when they reached 60%-70% confluence. The EO771 lifted cells were washed with PBS and suspended in fresh PBS to 5 × 10^5^ cells per 100 μL. The cells were kept on ice and 50 μL were injected into the 4th mammary gland. The 4T1-GFP-Luc lifted cells were washed with PBS and suspended in fresh PBS to 1 × 10^5^ cells per 50 μL. Tumors were measured every other day and resected when tumor volume reached approximately 1,000 mm^3^.

### Mammary tumor resection

Mice were sedated with a xylazine/ketamine (X:100 mg/kg; K:75 mg/kg) mix via intraperitoneal injection. Once the mice were fully sedated, the area was shaved and sterilized three times with iodine and alcohol. Skin incision was done with surgical scissors and the mammary tumors were removed using forceps and sterile scissors along with any visible lymph nodes. The incision was closed with polydioxanone sutures.

### Reserpine treatment

Mice with intramammary EO771 or 4T1 tumor sizes were monitored with a caliper and once tumors reached an approximate size of 30–50 mm^2^, we began pre-surgical treatment with reserpine (1 mg/kg dissolved in ascorbic acid and diluted in ddH2O) or vehicle (0.1% ascorbic acid diluted in ddH2O) three times (every other day) for one week. Once the tumor size measured approximately 200 mm^2^, we resected the tumors. One week following surgery, the mice were healed and started on weekly treatment with reserpine (or vehicle). When we observed animals displayed signs of respiratory stress or became moribund, we sacrificed the mice to collect the lungs to analyze the tissue for metastatic lesions.

Mice with intramammary EO771 or 4T1 tumors that were approximately 30–50 mm^2^ were housed with either reserpine chow (4 mg/kg) or control chow to consume ad libitum. Reserpine and Vehicle Chow were replaced every four days. Once the tumor size measured approximately 200 mm^2^, we resected the tumors. During suture recovery, the mice were given recovery gel food. One week after surgery or upon healed sutures, the animals resumed their reserpine or vehicle chow. When animals displayed signs of respiratory stress or became moribund, they were sacrificed and their lungs were analyzed for metastatic lesions.

### Paclitaxel treatment

Paclitaxel was dissolved in DMSO and then diluted in Tween 80, polyethylene glycol 300, ddH2O, and PBS to a final concentration of 2.5 mg/mL. The stock solution was aliquoted and frozen at −20 °C until needed. After determining mouse weight, the stock solution was further diluted in sterile PBS for intraperitoneal injection.

### Collection of lungs for H&E

Shortly following the euthanasia of the mice, the lungs were gently perfused with ice-cold PBS to wash out blood and prevent collapse of the lung architecture. We then perfused the lungs gently using 4% paraformaldehyde solution before processing for paraffin embedding.

### Analysis of tumor number and size in lung tissue

We used the Leica DM6000 widefield microscope that captured overlapping high-resolution, low-magnification images that then generated a composite image of the whole, H&E-stained lungs. The composite images were opened in ImageJ to remove noise and identify areas of metastasis for quantification. Composite images of H&E-stained primary tumors were used as a reference to set ImageJ parameters. We used ImageJ to create a mask that we superimposed on the original H&E pictures in order to verify areas that contained neoplasm cells. We used this mask over H&E overlap to count the number of nodules and to calculate the area of lung with tumor burden.

### Quantification and statistical analysis

The described results are representative of at least two independent experiments (*n* ≥ 4 mice per group unless specified otherwise). The analyses of all *in vitro* assays using cells or tissues from each of these animals were done at least in biological triplicates (which means samples from 3 tumors, 3 lungs, 3 spleens, *etc*). We presented the data as average ± S.E.M. Statistical analysis was performed using Microsoft Excel (Microsoft) or GraphPad Prism 7 software (GraphPad Prism Software, Inc.). The unpaired Student t-test was used for the comparison between the two groups. One-way ANOVA or two-way ANOVA analysis followed by the Bonferroni post-hoc test were used for the multiple comparisons. Repeated-measure two-way ANOVA (mixed-model) followed by the Bonferroni post-hoc test was used for the analysis of tumor growth curve. The Kaplan-Meier curves were used to depict the survival function from lifetime data for mice. A value of *P* < 0.05 was considered significant.

## RESULTS

### Loss of CH25H promoted breast cancer progression

**Figure 1.**
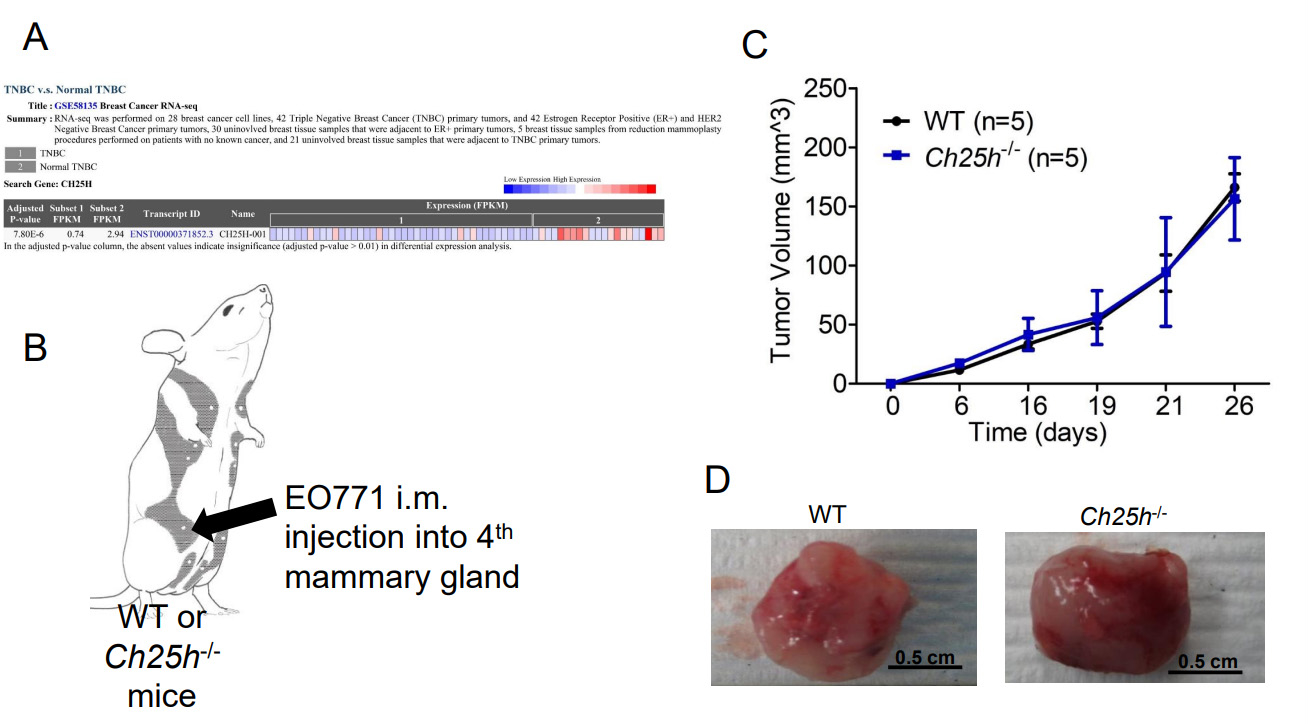
CH25H expression in Triple Negative Breast Cancer (TNBC) Primary Tumor. (A) Analysis of GSE58135 using GEO2R comparing CH25H expression between TNBC tissue and normal adjacent tissue (*n* = 42); (B) Schematic of EO771 intramammary (im) injection into wild-type (WT) and mice lacking CH25H (*Ch25h*^*−/−*^); (C) EO771 primary tumor growth following im injection overtime in WT and *Ch25h*^*−/−*^ mice (*n* = 5); (D) Representative images of excised primary EO771 tumors. Quantitative data are represented as mean ± SEM; *P* values: * *P* < 0.05; ** *P* < 0.01; *** *P* < 0.001 from Anova test. WT: wild-type.

The murine TNBC cell line EO771 releases TEVs that downregulate IFNAR1 in CD45+ cells that we isolated from WT mice spleens compared to PBS-treated cells or EVs from the immortalized mouse fibroblasts NIH-3T3s [Supplementary Figure 1]. This coincides with our previous data demonstrating that EO771 cells release TEVs that downregulate IFNAR1 in peripheral blood leukocytes (PBLs), splenocytes, and lung tissue, similar to TEVs from melanoma cells^[13]^. In our previous study, we demonstrated that loss of IFNAR1 following exposure to melanoma-derived TEVs resulted in loss of CH25H^[13]^. Therefore, we posited that loss of CH25H contributes to TNBC progression. Using GEO2R, we analyzed the publicly available RNAseq data set GSE58135 to compare CH25H expression in TNBC tumor tissue and normal adjacent tissue. CH25H expression was significantly lower in TNBC tissue compared to normal adjacent tissue [Figure 1A and Supplementary Table 1]. Considering normal adjacent tissue is not necessarily normal, we also used GEO2R to compare the TNBC tissue to normal tissue from reduction mammoplasty and again found that CH25H was downregulated in TNBC compared to normal tissue [Supplementary Table 2].

**Figure 2.**
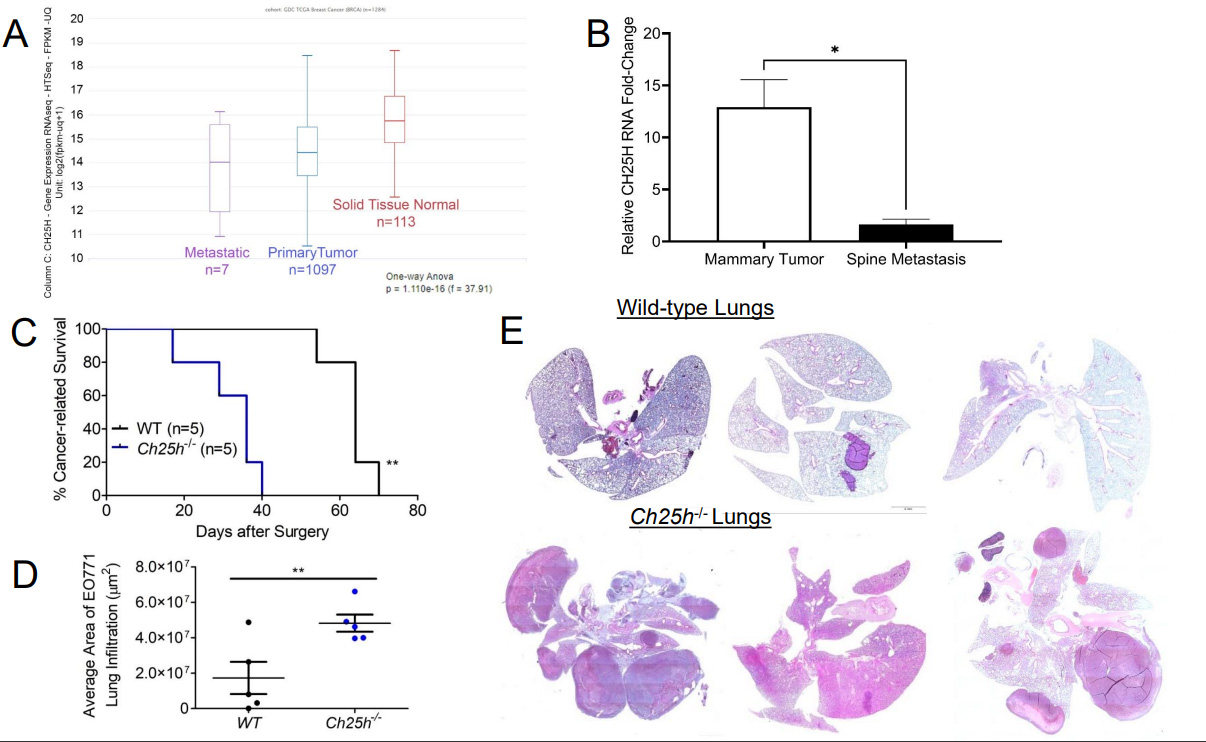
Loss of CH25H promotes distal metastasis in TNBC. (A) Analysis for CH25H expression from GDC and TCGA RNAseq data using UCSC Xena functional genomics explorer comparing normal tissue, primary tumor tissue, and metastatic tissue; (B) CH25H expression from GSE3797 RNAseq data set comparing 4T1.2 mammary tissue and corresponding spine metastatic tissue; (C) Kaplan-Meier curve comparing cancer-related survival of WT and *Ch25h*^*−/−*^ mice following EO771 primary tumor resection (*n* = 5); (D) Quantification of area affected by EO771 lung metastasis from H&E staining (*n* = 5); (E) Representative H&E staining of lungs from WT and *Ch25h*^*−/−*^ mice exhibiting respiratory distress after EO771 primary tumor resection. Quantitative data are represented as mean ± SEM; *P* values: * *P* < 0.05; ** *P* < 0.01; *** *P* < 0.001 and ns for not significant from Student’s t test (panels B and D), or log rank test (Panel C). TNBC: Triple-negative breast cancer; WT: wild-type; TCGA:The Cancer Genomic Atlas;

To determine the role of CH25H in the growth of primary TNBC tumors, we injected EO771 cells into the 4th mammary glands of wild-type (WT) and *Ch25h*^*−/−*^ mice [Figure 1B]. However, the tumors grew at the same rate and the tumors did not appear to escape the mammary capsule [Figure 1C]. Moreover, upon resection of the primary tumors, gross anatomy figures demonstrated that the tumors remained within the mammary capsule with no noticeable differences in vascularization or color [Figure 1D]. Considering clinical data associated CH25H expression with delayed development of metastasis, we set out to evaluate CH25H expression in metastatic lesions compared to primary breast cancer tumors and normal breast tissue. Using UCSC Xena Functional Genomics Explorer, we found that expression of CH25H was significantly lower in the metastasis samples compared to primary tumor and solid normal tissue [Figure 2A]^[24]^. To determine if this observation was observed in the 4T1 TNBC mouse model, we used the publicly available RNAseq data set GSE37975^[7]^. Data demonstrated that CH25H expression was lower in bone metastatic tissue compared to the 4T1 primary tumor [Figure 2B]. To demonstrate that loss of CH25H was a hallmark of distal TNBC metastasis, we followed WT and *Ch25h*^*−/−*^ following removal of the EO771 primary tumor. Twenty days after surgery, *Ch25h*^*−/−*^ mice began to exhibit difficulty breathing and were therefore sacrificed [Figure 2C]. By 40 days, all *Ch25h*^*−/−*^ mice had to be sacrificed due to signs of respiratory distress, which suggested pulmonary metastasis [Figure 2C]. However, the EO771 tumor-bearing WT mice survived significantly longer following surgery before exhibiting signs of respiratory distress [Figure 2C]. H&E analysis of the lung tissue demonstrated that *Ch25h*^*−/−*^ were more susceptible to aggressive lung metastasis compared to WT mice following removal of the EO771 tumor [Figure 2D and E]. This suggested that loss of IFNAR1 and subsequently CH25H in stromal cells contributes to distal metastasis.

#### Elevated IFN1 signaling in the host promotes TNBC tumor growth and metastasis

**Figure 3.**
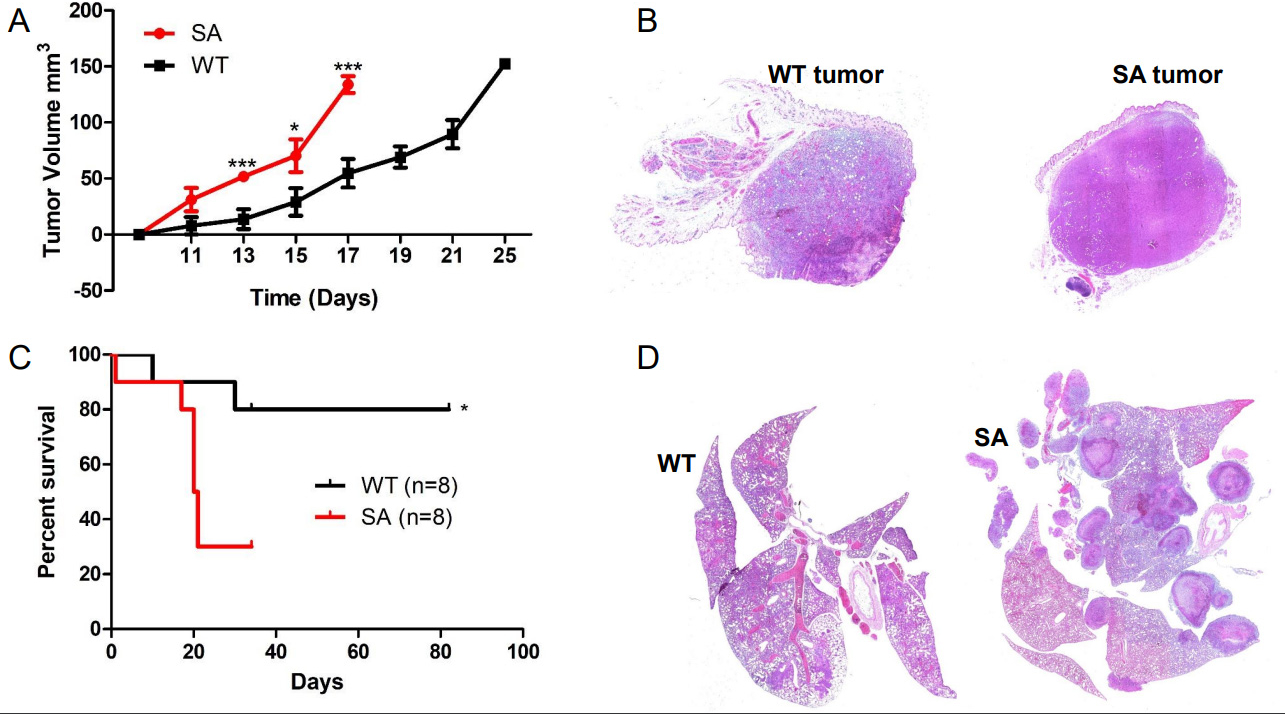
Sustained type 1 interferon signaling promotes TNBC cancer progression. (A) 4T1 primary tumor growth following im injection overtime in WT and SA knock-in mice (*n* = 8); (B) Representative images of excised primary 4T1 tumors; (C) Kaplan-Meier curve comparing cancer-related survival of WT and SA knock-in mice following 4T1 primary tumor resection (*n* = 8); (D) Representative H&E staining of lungs from WT and *Ch25h*^*−/−*^ mice exhibiting respiratory distress after EO771 primary tumor resection. Quantitative data are represented as mean ± SEM; *P* values: * *P* < 0.05; ** *P* < 0.01; *** *P* < 0.001 from Anova test. SA: Ifnar1S526A; WT: wild-type.

We initially anticipated that, like other cancer models, TNBC in the *Ifnar1*^S526A^ (SA) knock-in mouse would delay tumor growth and suppress the development of metastasis. However, 4T1 cells orthotopically injected into the 4th mammary gland of SA mice resulted in faster-growing tumors compared to 4T1 cells in WT [Figure 3A]. H&E staining demonstrated that the 4T1 tumors in the SA mice were dense with no adipose tissue or normal mammary gland tissue compared to 4T1 tumors from WT mice [Figure 3B]. When tumors reached 150 mm^3^, they were excised and mice were monitored for signs of metastasis. The SA mice developed pulmonary distress and exhibited paralysis in their legs much earlier than WT mice, resulting in poor overall survival [Figure 3C]. Analysis of the lung demonstrated severe metastasis in the SA mice following 4T1 excision compared to the WT mice [Figure 3D]. This suggested that IFN1 signaling in the host compartment accelerates TNBC tumorigenesis and metastatic development.

### Reserpine suppressed TNBC TEV release

**Figure 4.**
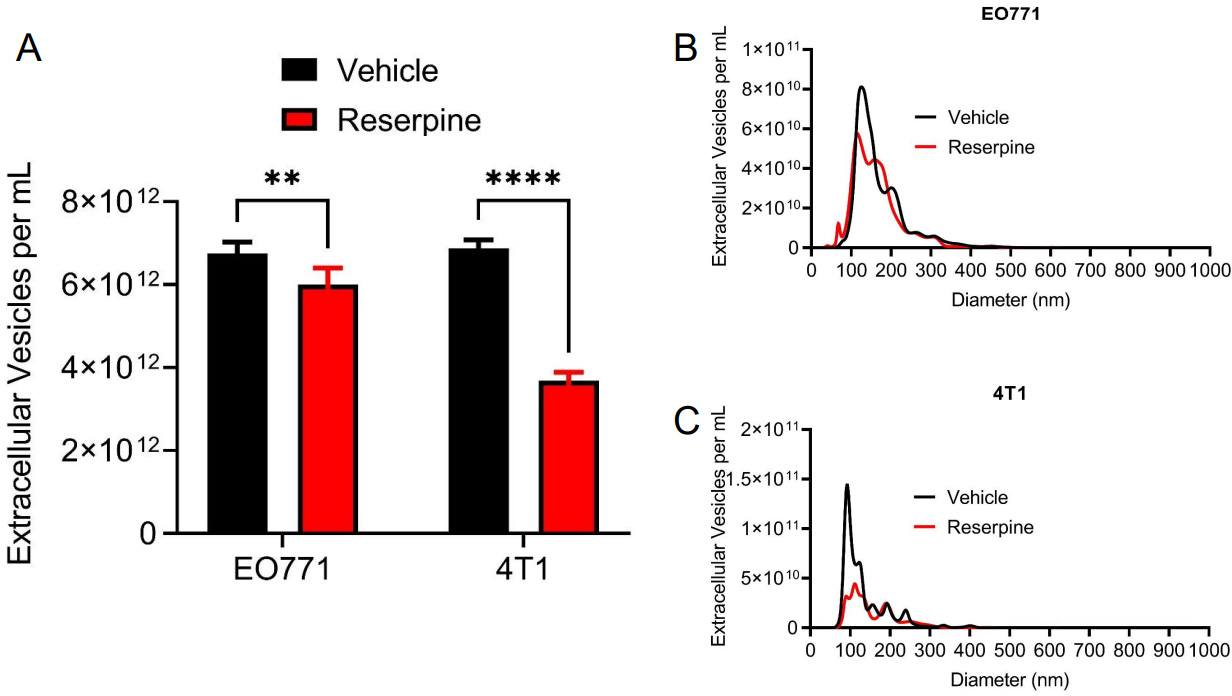
Reserpine suppresses release of TEV from murine TNBC cell lines. (A) Quantification of total extracellular vesicle concentration from EO771 and 4T1 cell culture media after 24 h in vehicle or 10 μM reserpine; (B) Nanoparticle tracking analysis comparing size distribution and concentration of TEV from EO771 cell culture media after 24 h in vehicle or 10 μM reserpine; (C) Nanoparticle tracking analysis comparing size distribution and concentration of TEV from 4T1 cell culture media after 24 h in vehicle or 10 μM reserpine. Quantitative data are represented as mean ± SEM; *P* values: * *P* < 0.05; ** *P* < 0.01; *** *P* < 0.001 and ns for not significant from Student’s t test (panel A).

We previously reported that reserpine limited the incorporation of TEVs *in vivo* and *in vitro* to overcome the lack of Ch25h expression^[13]^. Interestingly, we also observed that reserpine treatment decreased circulating TEV in B16F10 tumor-bearing mice and decreased TEV concentration in B16F10 cell culture media^[13]^. To demonstrate that reserpine can suppress TEV release by TNBC cell lines, an equal number of EO771 and 4T1-GFP cells were treated with vehicle or 10 μM reserpine for 24 h before collecting cell culture media for EV isolation via centrifugation. Nanoparticle tracking analysis (NTA) demonstrated that reserpine suppressed the release of TEVs into culture media in both EO771 and 4T1-GFP [Figure 4A]. NTA analysis further showed that cells treated with reserpine released TEVs within a similar size range as vehicle-treated cells [Figure 4B and C]. Interestingly, a comparison of the size distribution curves demonstrated that reserpine treatment affected the release of TEVs between 100 and 200 nm in diameter [Figure 4B and C].

**Figure 5.**
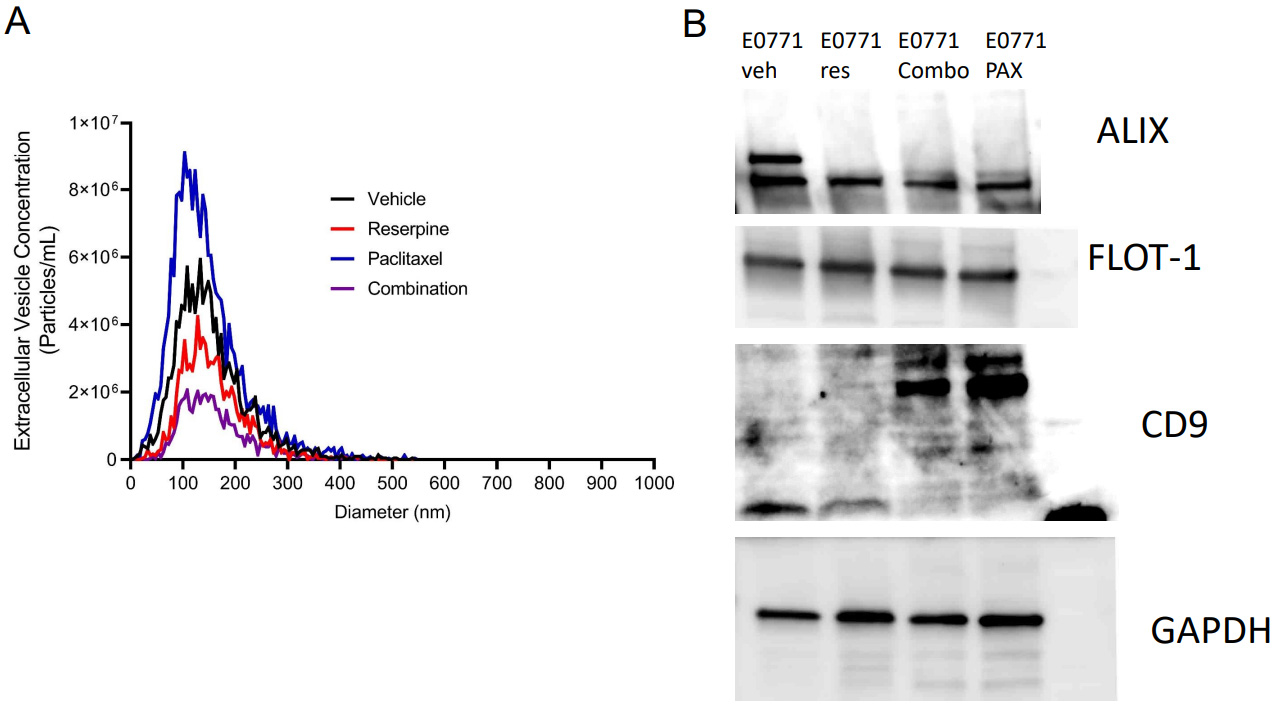
Reserpine suppresses release of paclitaxel-induced TEVs in TNBC. (A) Nanoparticle tracking analysis comparing size distribution and concentration of TEV from EO771 cell culture media after 24 h in vehicle, 10 μM reserpine, 100 μM paclitaxel, or both 10 μM reserpine with 100 μM paclitaxel (average of 2 separate collections); (B) Western blot analysis of the EV biomarkers ALIX, flotillin-1 (FLOT-1), CD9, and GAPDH from TEV isolated from EO771 cell culture media after 24 h in vehicle, 10 μM reserpine, 100 μM paclitaxel, or both 10 μM reserpine with 100 μM paclitaxel. TEVs: Tumor-derived extracellular vesicles; TNBC: Triple-negative breast cancer; ALIX: ALG-2-interacting protein X; CD9: Tetraspanin Cluster Domain 9 protein; GAPDH: Glyceraldehyde-3-phosphate dehydrogenase.

A previous study demonstrated that paclitaxel increased the release of TEVs from TNBC cells to promote metastasis^[5]^. To demonstrate that reserpine could suppress paclitaxel-induced TEV release, an equal number of EO771 cells were seeded and treated with vehicle, 10 μM reserpine, 100 μM paclitaxel, or both 10 μM reserpine with 100 μM paclitaxel for 24 h before culture media was collected for EV isolation. Like the 4T1 cells^[5]^, NTA demonstrated that paclitaxel increased release of TEVs from EO771 in culture [Figure 5A]. Cells treated with both reserpine and paclitaxel further decreased TEV release from EO771 cells [Figure 5A]. Western blot analysis demonstrated that EO771 cells treated with vehicle release TEVs with two forms of ALIX [Figure 5B]. Flotillin-1 (FLOT-1) expression was unchanged in TEVs from all treatment groups [Figure 5B]. EO771 cells treated with paclitaxel or with both paclitaxel and reserpine release TEVs with more CD9 compared to cells treated with vehicle or reserpine alone [Figure 5B]. To confirm that our isolation method resulted in isolation of EVs, we used Western blot analysis to demonstrate presence of CD63 and GAPDH but not the Golgi marker GM130 [Supplementary Figure 2]. This data suggests that reserpine could suppress release of paclitaxel-mediated TEV release to suppress TNBC metastasis.

### Inhibiting TNBC EV function with reserpine suppressed lung metastasis

**Figure 6.**
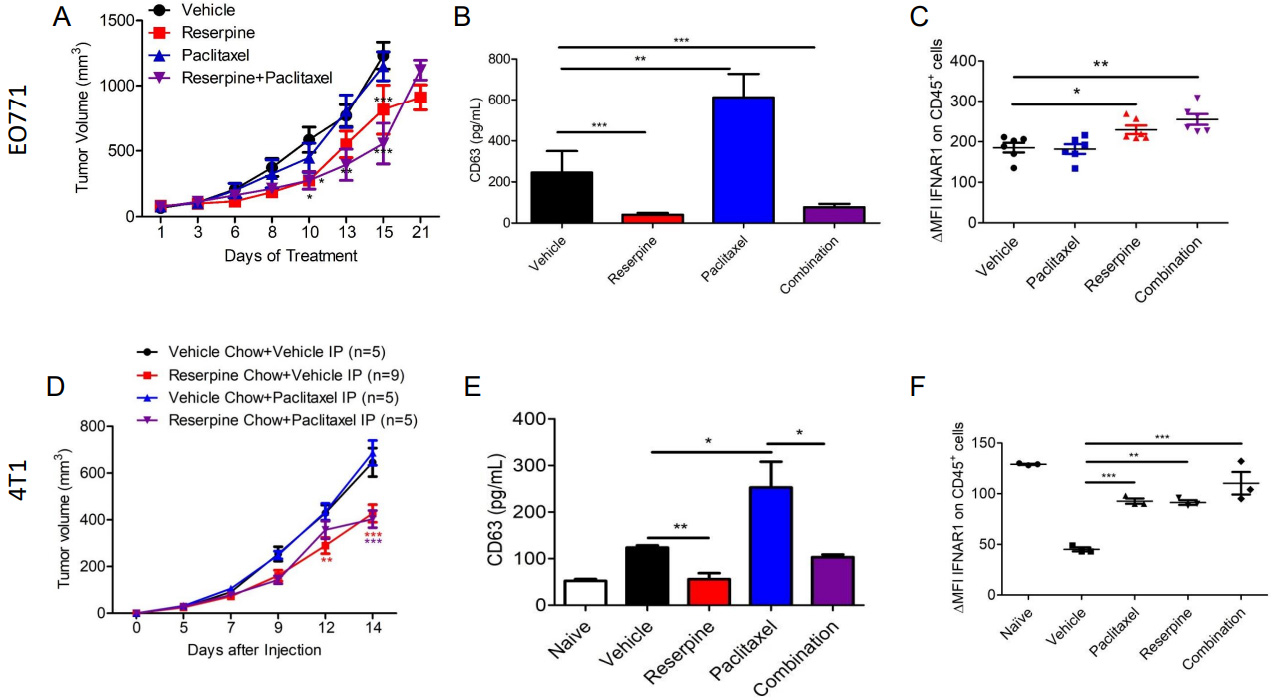
Suppressing TEV release delays tumor growth and prevents loss of IFNAR1. (A) Tumor growth curve of EO771 tumor-bearing WT mice treated intravenously (iv) with vehicle, 1 mg/kg of reserpine (every other day), 2 mg/kg paclitaxel (twice a week), or both reserpine and paclitaxel (*n* = 5, each group); (B) ELISA for CD63 to quantify TEVs from serum of WT mice treated intravenously (iv) with vehicle, 1 mg/kg of reserpine (every other day), 2 mg/kg paclitaxel (twice a week), or both reserpine and paclitaxel with EO771 with tumors of similar size; (C) Flow cytometry analysis comparing change in mean fluorescent intensity (ΔMFI) of IFNAR1 in CD45+ cells from peripheral blood of WT mice with EO771 with tumors of similar size treated intravenously (iv) with vehicle, 1 mg/kg of reserpine (every other day), 2 mg/kg paclitaxel (twice a week), or both reserpine and paclitaxel (*n* = 5); (D) Tumor growth curve of 4T1-GFP tumor-bearing WT mice treated intravenously (iv) with vehicle and given vehicle chow (*n* = 5), iv vehicle and reserpine chow (*n* = 9), iv injection with 2 mg/kg paclitaxel (twice a week) and vehicle chow (*n* = 5), or iv injection with paclitaxel and reserpine chow (*n* = 5); (E) ELISA for CD63 to quantify TEVs from serum of WT mice with similar size tumor from (D); (F) Flow cytometry analysis comparing change in mean fluorescent intensity (ΔMFI) of IFNAR1 in CD45+ cells from peripheral blood of WT mice with EO771 with tumors of similar size from (D); Quantitative data are represented as mean ± SEM; *P* values: * *P* < 0.05; ** *P* < 0.01; *** *P* < 0.001, and ns for not significant from Anova test (panels A and B) or Student’s t test (panels B, C, E, and F).

Previous studies demonstrated that reserpine prevents loss of IFNAR1 and CH25H expression in melanoma to suppress the development of metastasis^[13]^. To determine if reserpine can mimic IFNAR1/CH25H/25HC function to suppress TNBC distal metastasis, we injected EO771 into the 4th mammary of WT C57BL/6 mice and 4T1-GFP into the 4th mammary of WT Balb/c mice. EO771 tumor-bearing mice were divided into four groups receiving intraperitoneal (ip) injections of vehicle, 1 mg/kg of reserpine (every other day), 2 mg/kg paclitaxel (twice a week), or both reserpine and paclitaxel. 4T1-GFP tumor-bearing mice were also divided into 4 groups receiving ip injections of vehicle and given normal chow or reserpine chow (4 mg/kg) ad libitum, or receiving ip injections of 2 mg/kg paclitaxel and given normal chow or reserpine chow ad libitum. Paclitaxel treatment did not affect EO771 or 4T1-GFP tumor growths compared to vehicle [Figure 6]. However, reserpine alone or in combination with paclitaxel significantly delayed the growth of both EO771 and 4T1-GFP tumors [Figure 6].

Plasma was isolated from the blood of tumor-bearing mice with tumors of similar size to examine changes in circulating TEVs using anti-CD63 ELISA. We demonstrated that paclitaxel treatment in both EO771 and 4T1-GFP tumor-bearing mice resulted in increased CD63 levels compared to mice receiving vehicle [Figure 6]. This coincides with a study that showed paclitaxel increases the release of TEVs from TNBC cells^[5]^. As observed in melanoma-bearing mice^[13]^, EO771 and 4T1-GFP tumor-bearing mice receiving reserpine treatment also exhibited less CD63, and therefore less TEVs, in their plasma [Figure 6]. Interestingly, reserpine was also able to limit paclitaxel-induced TEV release in the plasma of tumor-bearing mice[Figure 6]. This observation was confirmed via NTA using TEVs isolated from the remaining serum from vehicle- and reserpine-treated EO771 tumor-bearing mice [Supplementary Figure 2A]. Western blot analysis was performed to further characterize the isolated TEVs from serum [Supplementary Figure 2B]. Interestingly, TEVs isolated from the serum of 4T1-GFP tumor-bearing mice on reserpine chow carried less FLOT-1, and ALIX and GAPDH with post-translational modifications [Supplementary Figure 2B]. These changes in TEV protein content were not present in TEVs isolated from EO771 tumor-bearing mice [Supplementary Figure 2B]. This may be the difference between intravenous reserpine administration and ingestion of reserpine in chow.

We have previously demonstrated that reserpine was able to suppress the loss of IFNAR1 in peripheral blood leukocytes in tumor-bearing mice^[13]^. Analysis of CD45+ peripheral blood leukocytes (PBLs) isolated from EO771 and 4T1-GFP tumor-bearing mice with tumors of similar size demonstrated that reserpine treatment prevented loss of IFNAR1 [Figure 6]. Moreover, reserpine also suppressed the loss of IFNAR1 on PBLs in mice that were also given paclitaxel [Figure 6].

**Figure 7.**
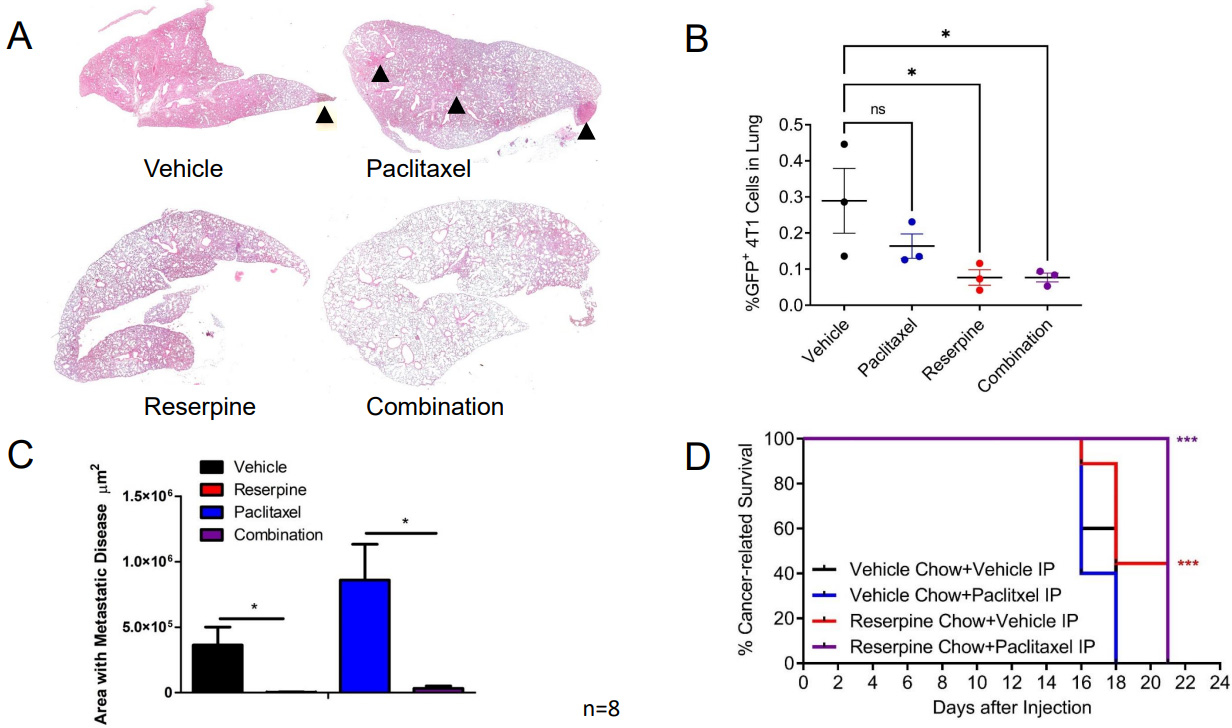
Suppressing TNBC TEV release suppresses pulmonary metastasis and improves cancer-related survival. (A) Representative H&E staining from Balb/c WT mice following 4T1-GFP+ tumor resection receiving designated treatment; (B) Quantification of lung area with 4T1-GFP metastasis from mice treated intravenously (iv) with vehicle and given vehicle chow, iv vehicle and reserpine chow, iv injection with 2 mg/kg paclitaxel (twice a week) and vehicle chow, or iv injection with paclitaxel and reserpine chow (*n* = 8, each group); (C) Flow cytometry analysis of dissociated lung tissue from mice to detect GFP+ cells from WT mice after 4T1-GFP tumor resection exhibiting decreased health condition (*n* = 3, each group). (D) Kaplan-Meier curve comparing cancer-related survival of WT mice following 4T1-GFP tumor resection treated intravenously (iv) with vehicle and given vehicle chow, iv vehicle and reserpine chow, iv injection with 2 mg/kg paclitaxel (twice a week) and vehicle chow, or iv injection with paclitaxel and reserpine chow (*n* = 8, each group). Quantitative data are represented as mean ± SEM; *P* values: * *P* < 0.05; ** *P* < 0.01; *** *P* < 0.001, and ns for not significant from Student’s *t* test (panels B and C) or log rank test (D). TEVs: Tumor-derived extracellular vesicle; TNBC: Triple-negative breast cancer; WT: wild-type mice; 4T1-GFP: GFP expressing 4T1 murine breast cancer cell line.

To determine if this decrease in circulating TEVs and retention of IFNAR1 mediated by reserpine treatment can also limit paclitaxel-induced metastasis, we resected the 4T1-GFP tumors from the groups receiving ip injections of vehicle and given normal chow or reserpine chow (4 mg/kg) ad libitum, or receiving ip injections of 2 mg/kg paclitaxel and given normal chow or reserpine chow ad libitum. Following surgery, the mice were placed back on their designated chows and received weekly ip injections of vehicle or paclitaxel. Analysis of H&E staining demonstrated that mice on vehicle and paclitaxel eating the vehicle chow developed metastasis [Figure 7A and B]. This was confirmed by dissociating the lungs of three mice from each group for flow cytometry [Figure 7C]. As such, mice receiving reserpine treatment alone or in combination with paclitaxel exhibited better overall survival compared to mice receiving paclitaxel alone or vehicle alone [Figure 7D].

### NUPR1 drives TEV-mediated cancer progression in TNBC

**Figure 8.**
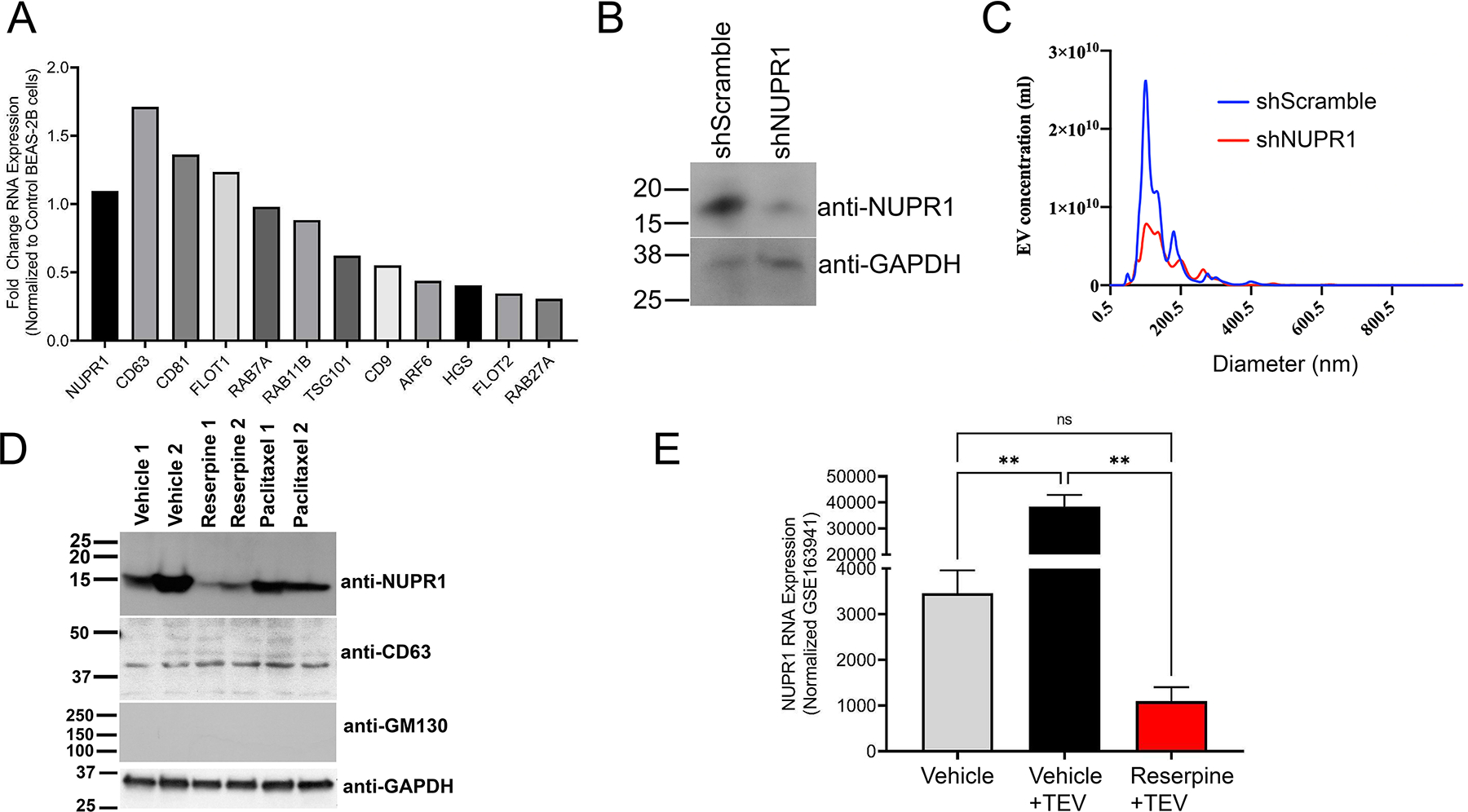
NUPR1 increases TEV release and packages NUPR1 in TEVs to promote cancer progression. (A) Western blot analysis for NUPR1 using 10 μg of EO771 TEVs isolated from 2 separate collections 24 hrs after cells were treated with vehicle, 10 μM reserpine, or 100 μM paclitaxel; (B) RNAseq analysis of EV markers using RNA from normal human lung bronchial epithelial cells BEAS-2B and BEAS-2B engineered to overexpress NUPR1; (C) Nanoparticle tracking analysis of TEVs from nickel transformed BEAS-2B cells and nickel transformed BEAS-2B cells expressing shRNA for NUPR1; (D) Western blot analysis for NUPR1 in EVs from nickel transformed BEAS-2B cells and nickel transformed BEAS-2B cells expressing shRNA for NUPR1. The EVs were further characterized for EV markers CD63 and GAPDH and the non EV biomarker GM130; (E) NUPR1 RNA expression from mouse *Ch25h*^*−/−*^ endothelial cells treated with vehicle, TEV, or TEV with 10 μM reserpine. TEVs: Tumor-derived extracellular vesicles; NUPR1: Nuclear protein 1; EV: Extracellular vesicle.

To determine role of NUPR1 in extracellular vesicle biogenesis and incorporation, we overexpressed NUPR1 in the immortalized, normal human bronchial alveolar cell line BEAS-2B and used the RNA for RNAseq [Figure 8A]. NUPR1 overexpression resulted in upregulation of various extracellular vesicle markers associated with extracellular vesicle biogenesis and incorporation [Figure 8A]. Western blot analysis determined that EVs from nickel-transformed BEAS-2B cells carry NUPR1 and that nickel-transformed cells expressing shRNA targeting NUPR1 release EVs without NUPR1 [Figure 8B]. Moreover, nanoparticle tracking analysis demonstrated that knockdown of NUPR1 also decreased release of extracellular vesicles [Figure 8C]. As we previously demonstrated that reserpine suppressed release of EVs from EO771 cells [Figure 4A]. Western blot analysis demonstrated that reserpine also suppressed NUPR1 cargo in EO771 extracellular vesicles [Figure 8D]. To demonstrate that NUPR1 presence in EVs is not limited to TNBC cells, we analyzed NUPR1 expression in EVs from another cancer type. Like breast cancer, colon cancer metastasis is also driven by elevated NUPR1^[25]^. Western blot analysis demonstrated that murine colon adenocarcinoma cell line MC38 released EVs that also carry NUPR1 [Supplementary Figure 3]. Similar to what is observed in the EO771-derived EVs, MC38 cells treated with reserpine resulted in the release of EVs with less NUPR1 protein [Supplementary Figure 3]. Interestingly, RNAseq analysis further demonstrated that TEVs from MC38 cells upregulated NUPR1 in endothelial cells isolated from *Ch25h*^*−/−*^ mice, but reserpine suppressed the NUPR1 upregulation induced by the TEVs [Figure 8E].

## DISCUSSION

Studies have demonstrated that IFN1 signaling induces the expression of pro-apoptotic, anti-angiogenic, and immunomodulatory genes that could be used in therapy against cancers and infectious diseases. In randomized trials, administration of IFN1 ligands resulted in the upregulation of estrogen receptors (ERs) in breast cancer tumor cells that would make them sensitive to ER-targeted therapies^[26]^. However, examination of IFN1 signaling in cancer cells is insufficient to determine breast cancer progression. Lamsal *et al.* observed increased IFN1 signaling in metastatic cancer cell lines, yet when implanted to form primary tumors, the tissue exhibited dampened IFN1 signaling^[27]^. Weichselbaum *et al.* analyzed breast cancer tissue following radiation and chemotherapy and observed upregulation of interferon-related DNA damage resistant signature (IRDS) that contributed to the development of metastasis^[28]^. However, the patient samples were predominantly ER-positive, which suggests IFN1 signaling alone may not be predictive of metastasis. In fact, Doherty *et al.* demonstrated that IFN1 signaling inhibited the development of cancer stem cell properties in TNBC cells^[10]^. Moreover, it is also important to examine the status of IFN1 signaling in the stromal compartment and how that contributes to TNBC progression to metastasis.

Previous publications have used the *Ifnar1*^−/−^ mouse to demonstrate that loss of IFN1 signaling in the stromal compartment promotes TNBC metastasis^[7,14]^. Decreased expression of IFN1-induced genes in breast cancer biopsies correlates with poor patient outcomes^[27]^. IFN1 signaling can be dampened by affecting STAT1 activation and IFNAR1 cell surface expression^[13,29,30]^. Murine TNBC cells release TEVs that downregulate IFNAR1 in recipient cells [Supplementary Figure 1C] and distal tissues^[13]^. Loss of IFNAR1 following exposure to TEVs results in the development of pre-metastatic niches to promote metastasis via the downregulation of CH25H expression^[13]^. CH25H expression is lower in TNBC tissue compared to normal tissue [Figure 1A]. Measurement of oxysterols in serum from breast cancer patients demonstrated that levels of 25HC, the product of CH25H, were lower compared to all other circulating oxysterols measured^[31]^. As such, the observed metastasis may be correlated to dampened IFN1 signaling via downregulation of CH25H. Analysis of human TNBC data demonstrated that CH25H levels are lower in metastatic tissue compared to primary tumors and more so compared to normal adjacent tissue [Figure 2A]. Murine TNBC primary tumors within the mammary gland grew at similar rates between WT and *CH25H*^*−/−*^ mice [Figure 1B–D]. However, mice lacking CH25H exhibited poor survival following primary tumor resection and developed more severe metastatic lesions compared to WT mice [Figure 2C–E]. This coincides with many studies demonstrating the role of IFN1 signaling as anti-tumorigenic and anti-metastatic. However, studies have also demonstrated the detrimental effects of sustained IFN1 signaling in breast cancer^[15,16]^. Indeed, our SA knock-in model demonstrated that sustained IFN1 signaling via an IFNAR1 that cannot be downregulated accelerated tumor growth, worsened overall survival, and generated more severe lung metastasis[Figure 3]. This suggested an IFN1-independent mechanism.

TEVs contribute to the generation of pre-metastatic niche^[13,23]^, the development of distal metastasis^[13,23]^, and resistance to therapy^[32–34]^. Studies have demonstrated that paclitaxel and other neoadjuvant chemotherapies work can be critical in suppressing the progression of invasive breast cancers^[4–6,15]^. However, as patients continue their therapy, they develop resistance^[4–6,15]^. Acquired resistance to chemotherapy can be due to changes in the malignant cancer cells or in the stromal environment that may promote the development of metastatic disease. Paclitaxel treatment results in the release of pro-metastatic TEVs in murine TNBC models. This suggests that TNBC patients undergoing chemotherapy may develop resistance through increased levels of TEVs in circulation contributing to loss of the IFNAR1/CH25H/25HC pathway. Therefore, finding pathways and medications that can suppress TEV release and/or uptake may improve the efficacy of chemotherapeutic agents and suppress the development of distal metastasis.

Reserpine suppressed the release of TEVs from TNBCs in culture [Figure 4A–C]. As previously observed, treatment with paclitaxel increased TEV release [Figure 5A]. Interestingly, reserpine suppressed paclitaxel-induced TEV release *in vitro* [Figure 5A]. Interestingly, analysis of EVs from 4T1 tumor-bearing mice demonstrated that the reserpine treatment resulted in EVs with less flotillin-1 (FLOT-1) and GAPDH bands of larger or smaller size compared to the other treatment conditions [Supplementary Figure 2B]. While we currently do not understand how reserpine could affect GAPDH post-translational modification status in the released EVs, our data indicate that reserpine affects NUPR1 expression and, in turn, FLOT-1. This may account for the diminished protein levels of FLOT-1 in EVs isolated from the serum of 4T1 tumor-bearing mice receiving reserpine [Figure 2B].

Reserpine alone and in combination with paclitaxel delayed TNBC tumor growth, decreased TEVs in circulation, and prevented loss of IFNAR1 in CD45+ cells in TNBC tumor-bearing mice [Figure 6]. As previously observed, paclitaxel increased the incidence of lung metastasis in TNBC tumor-bearing mice [Figure 7A–C], likely through increased circulating TEVs. However, when TNBC tumor-bearing mice were treated with paclitaxel in combination with reserpine, the development of lung metastasis was suppressed and overall survival improved [Figure 7].

Elevated NUPR1 expression in TNBC is associated with poor overall survival, increased metastasis, and resistance to chemotherapy^[19–21]^. Treatment with chemotherapy drugs induces NUPR1 expression^[20]^. Similarly, studies have demonstrated that chemotherapy agents increase the release of prometastatic TEVs^[5]^. Overexpression of NUPR1 in non-cancer cells upregulated genes associated with extracellular vesicle biogenesis and incorporation [Figure 8A]. TNBC cells and other cancer cells package NUPR1 in TEVs [Figure 8B and D, Supplementary Figure 3]. Knockdown of NUPR1 suppressed packaging of NUPR1 into TEVs but also decreased TEV release [Figure 8C]. Reserpine treatment functioned like shNUPR1 by limiting TEV biogenesis and suppressing NUPR1 packaging into TEVs [Figure 8D and Supplementary Figure 3]. Moreover, analysis of RNAseq data of primary endothelial cells from Ch25h null mice treated with vehicle or reserpine and then treated with EVs from the colon cancer cell line MC38, which also release EVs with NUPR1 [Supplementary Figure 3A], demonstrated that cells treated with NUPR1-rich EVs upregulated NUPR1 expression while reserpine suppressed NUPR1 expression [Figure 8E]. While discontinued, we have presented evidence that low doses of reserpine can suppress TEV-mediated cancer progression in TNBC murine mouse models by altering NUPR1 expression.

As our data and previous publications have demonstrated, reserpine affects the release of TEVs as well as the transcription factor NUPR1 and IFN1 signaling. However, the effects of reserpine on other components secreted by cancer cells, such as cytokines and chemokines (i.e., the secretome), is yet to be determined. Our attempts to determine the role of EV-depleted conditioned media in cancer progression independently or in concert with TEVs have yielded variable results that require efforts beyond the scope of this work to resolve. As such, it would be prudent to investigate other components of the secretome that work in concert or independently of TEVs. As the soluble component of cancer cell secretomes includes proteases and ligands that may bind to cell surface proteins on the TEVs, we hope to optimize methods of protein isolation and characterization. Such an endeavor may provide insight into how to further alter TEV composition and intervene in TEV-mediated cancer progression.

## CONCLUSION

In this study, we present data that loss of the IFN1 ISG CH25H promotes metastasis in TNBC as observed in other cancers^[13,35]^, and that hyperactive IFN1 signaling using the SA mouse model also promotes TNBC metastasis to the lung. Though both diminished and hyperactive IFN1 signaling promoted TNBC metastasis, suppression of EV release using reserpine delayed tumor growth and prevented loss of IFNAR1 in stromal cells even in the presence of paclitaxel. By suppressing the release of EVs in tumor-bearing mice, we suppressed the development of lung metastasis and improved overall survival. Studies have demonstrated that estrogen receptor-positive breast cancer cells, and TNBC cell lines and tissue have elevated levels of NUPR1^[20,21]^. NUPR1 is often studied for its role as a transcription factor. However, our data demonstrated the presence of NUPR1 in cancer cell-derived EVs. Therefore, cytoplasmic NUPR1 may participate in loading EVs with their unique cargo. Moreover, early-stage TNBC patients undergo chemotherapy with other chemotherapeutic agents, such as doxorubicin and 5-fluorouracil. In some cases, a combination of chemotherapy agents is administered. As such, further investigation into the role of NUPR1 in EV biogenesis, packaging, and release following combination therapy may provide novel targets for improving TNBC therapy.

## Supplementary Material

Supplementary Material

## Figures and Tables

**Table 1.** List of mice, cell lines, reagents, plasmids, antibodies, and software

Mouse monoclonal anti-IFNAR1-PE (clone MAR1-5A3)	BioLegend	Cat#127311; RRID: AB_1134011
Mouse monoclonal anti-CD45-APC (clone 30-F11)	BioLegend	Cat#103112; RRID: AB_312977
Alix (3A9) Mouse monoclonal	Cell Signaling Technology	Cat#2171
GM130 (D6B1) XP Rabbit monoclonal	Cell Signaling Technology	Cat#21480
Flotillin-1 (D2V7) XP Rabbit monoclonal	Cell Signaling Technology	Cat#18634
CD9 (D8O1A) Rabbit monoclonal	Cell Signaling Technology	Cat#13174
GAPDH (D4C6R) Mouse monoclonal	Cell Signaling Technology	Cat#97166
CD63 Rabbit monoclonal	ABclonal	Cat#A19023
p8 (aka NUPR1) shRNA Plasmid expressing nickel-transformed BEAS-2B cells		Provided by Max Costa^[22]^
Control shRNA plasmid expressing nickel-transformed BEAS-2B cells		Provided by Max Costa^[22]^
Lipofectamine 2000	Invitrogen	Cat#11668027
pCMV-NUPR1	Origene	Cat#RC222237
pCDNA3.1-Flag	Addgene	Cat#20011
Reserpine	Sigma-Aldrich	Cat#83580 CAS#50-55-5
Paclitaxel	Selleckchem	Cat# S1152 CAS# 918505-84-7
Reserpine Chow (4 mg reserpine/kg in Purina Rodent Chow)	Research Diets, Inc.	Cat# C18112901
Control Chow (Purina Rodent Chow)	Research Diets, Inc	Cat# C11000
Collagenase II	MP Biomedicals	Cat#1005002 CAS#9001-12-1
Collagenase D	Roche Diagnostics	Cat#11088882001
DNaseI, grade II	Roche Diagnostics	Cat#10104159001
Invitrogen Total Exosomes Isolation Reagent (from serum)	ThermoFisher Scientific	Cat# 4478360
Mouse CD63 Antigen (CD63) ELISA Kit	CUSABIO	Cat# CSB-EL004950MO
Mouse: EO771 mammary adenocarcinoma	CH3 Biosystems	Cat#940001
Mouse: MC38 murine colon adenocarcinoma	Millipore Sigma	SCC172
BEAS-2B	ATCC	Cat#CRL-3588
Mouse WT C57BL/6	The Jackson Laboratory	Cat#000664
Mouse: C57BL/6 Ch25h−/−	The Jackson Laboratory	Cat#016263
Mouse WT BALB/c	The Jackson Laboratory	Cat#000651
Mouse SA BALB/C	Generated in Fuchs Laboratory	
FlowJo software version 10	FlowJo Software	https://www.flowjo.com/
GraphPad Prism software	GraphPad software	http://www.graphpad.com
Microsoft Excel 2010	Microsoft Office	

## Data Availability

Not applicable.
