# Supplementary material for: NUPR1 packaged in extracellular vesicles promotes murine triple-negative breast cancer in a type 1 interferon-independent manner
Source: Extracell Vesicles Circ Nucl Acids. Author manuscript; Available in PMC 2024 Feb 23. (PMC10887431; doi:10.20517/evcna.2023.59)
Supplement: Supplementary Material [file NIHMS1960879-supplement-Supplementary_Material.zip › 202359-SupplementaryMaterials.pdf]

Supplementary Materials

NUPR1 packaged in extracellular vesicles promotes murine triple-negative breast cancer in a type 1 interferon-independent manner

Angelica Ortiz[1,2,*], Aikaterini Stavrou[1], Shan Liu[1], Danqi Chen[1], Steven S. Shen[3], Chunyuan Jin[1]

[1]Department of Medicine, Division of Environmental Medicine, New York University Grossman School of Medicine, New York, NY 10010, USA.

[2]Department of Biomedical Science, School of Veterinary Medicine, University of Pennsylvania, Philadelphia, PA 19104, USA.

[3]Clinical Translational Science Institute, University of Minnesota, Minneapolis, MN 55455, USA.

***Correspondence to:** Angelica Ortiz, PHD, Department of Medicine, Division of Environmental Medicine, New York University Grossman School of Medicine, 341 East 25th Street, New York, NY 10010, USA. E-mail: angelic.ortiz@gmail.com

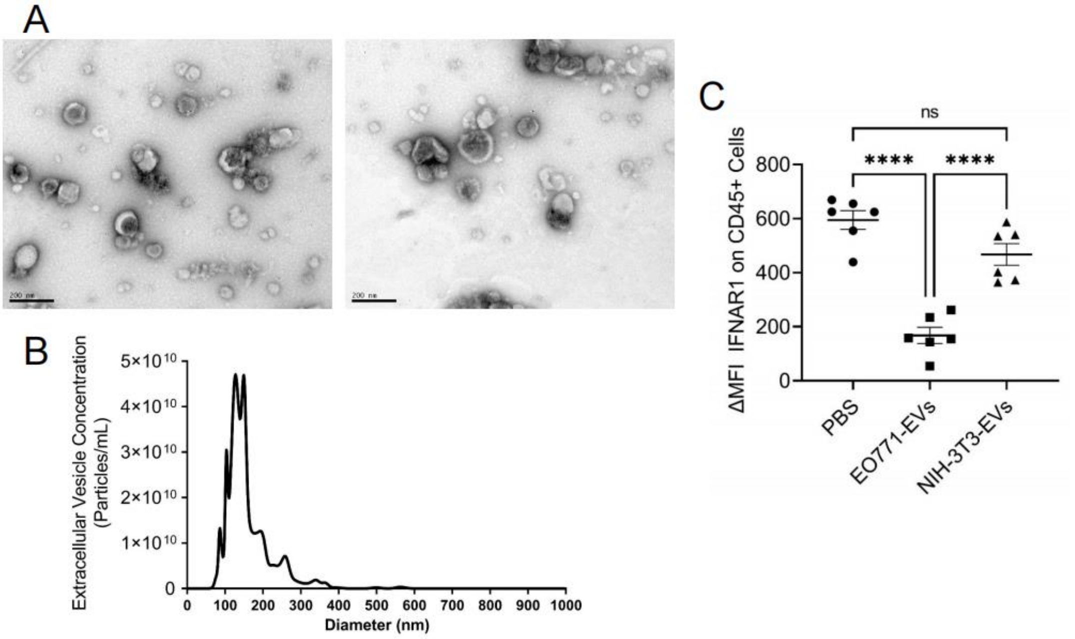

Supplemental Figure 1. EO771 cells release extracellular vesicles that downregulate

IFNAR1. (A) Transmission electron microscopy images of TEVs following isolation via ultracentrifugation. (B). Nanoparticle tracking analysis demonstrating TEV size distribution and concentration of size populations. (C). Flow cytometry analysis comparing change in mean fluorescent intensity (ΔMFI) of IFNAR1 in CD45+ cells from spleens following treatment with PBS, extracellular vesicles from EO771 (EO771-EVs), or extracellular vesicles from NIH-3T3 (NIH-3T3-EVs). Quantitative data are represented as mean ± SEM; p values: * $p<0.05$; ** $p<0.01$; *** $p<0.001$, and ns for not significant from Student's t test (panel C)

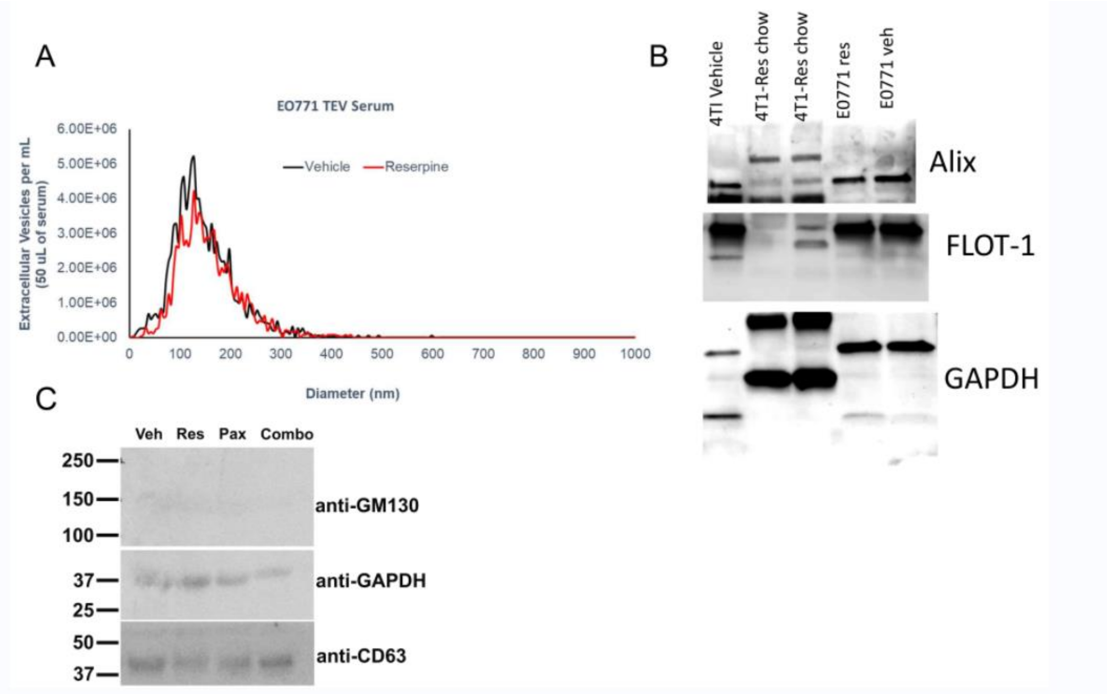

Supplemental Figure 2. Characterization of TNBC TEVs isolated from tumor bearing mice. (A) Nanoparticle tracking analysis of EO771 TEVs isolated from the serum of WT mice bearing tumors of similar size receiving intravenous treatment with vehicle or 10 µM reserpine. (B) Western blot analysis to compare EV biomarkers from TEV isolated from mice with similar size tumor receiving the designated treatment. (C) Western blot analysis of the EV biomarkers CD63 and GAPDH, and the non EV biomarker GM130 from TEV isolated from EO771 cell culture media after 24 hrs in vehicle, 10 µM reserpine, 100 µM paclitaxel, or both 10 µM reserpine with 100 µM paclitaxel.

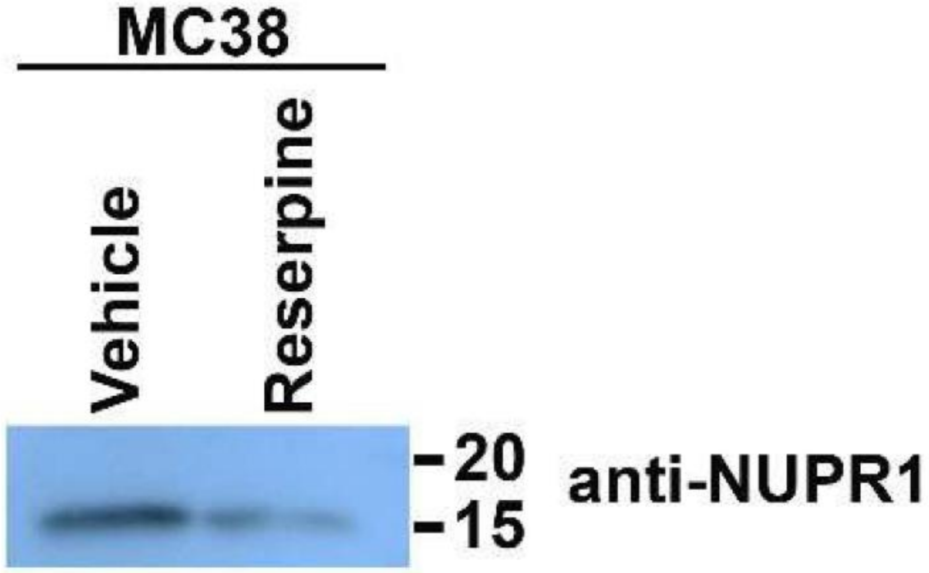

Supplemental Figure 3. MC83-derived TEVs carry NUPR1. Western blot analysis for NUPR1 using 10 µg of TEVs from MC38 cells treated with Vehicle or 10 µM reserpine for 24 hrs.

Supplemental Table 1. Significant Gene Expression Changes Between TNBC and non-TNBC tissue. List of significant changes in gene expression between TNBC and adjacent normal tissue from the GSE58135 dataset generated by GEO2R. CH25H highlighted in yellow.

Supplemental Table 2. Significant Gene Expression Changes Between TNBC and normal mammary tissue following reduction mammoplasty. List of significant changes in gene expression between TNBC and normal tissue from the GSE58135 dataset generated by GEO2R. CH25H highlighted in yellow.
